# The Recognition of Nystagmus

**Published:** 1904-04-23

**Authors:** 


### THE RECOGNITION OF NYSTAGMUS.

A mere book knowledge of medicine would convey the idea that the recognition and appreciation of the phenomena of disease offer no particularly serious difficulties. Symptoms or physical signs, it would appear, either exist or they do not exist, and an inference, one way or the other, follows the event. But the chastening hand of experience shows the art of clinical observation to be in practice much less simple than is suggested by its theoretical exposition. This is due in part to the fact that many of the evidences of disease are departures from the normal, not in kind but in degree. The physiological passes by almost inappreciable gradations into the pathological, and it often needs not only observation but experience and judgment to say with confidence when the limit has been definitely passed. Extreme departures are to be recognised even by the wayfaring man. But something more than this is aimed at by the physician. Hence the necessity for the constant observation of presumably healthy parts and their activities so that a confident appreciation of the physiological range may be secured. These principles receive an illustration in connection with the use of the term nystagmus. In some cases rhythmical oscillation of the eyeballs is manifest even to the unobservant. In a larger group it is only exerted when the eyeballs are forced into some special position as, for example, into extreme lateral deviation. It is here that difficulty arises, for it is beyond question that in many cases free from all suggestion of nervous disease such a position of the eyeballs excites more or less unsteadiness or even a definite manifestation of jerking movements. The question thus comes to be when such movements ought to be termed nystagmus, in the sense that there exists an undoubted pathological condition. It is not easy to suggest any certain rule regarding the line of distinction between the normal and the abnormal, but the point of importance is that this difficulty should be recognised and that when any possible doubt exists the facts themselves should be described rather than that their ambiguous significance should be concealed by the use of a term of unquestioned pathological import. That some physicians apply the term nystagmus in conditions where others would repudiate the use of the word is certain, and hence arise a number of actively contradictory statements. A series of sustained rhythmical oscillations is, of course, beyond dispute, but short of that it is at least open to question whether the pathological area has been certainly entered. And on general principles it is no misfortune to cultivate the description of facts rather than the adjustment of names.

